# Geospatial Analysis of Multilevel Socioenvironmental Factors Impacting the *Campylobacter* Burden among Infants in Rural Eastern Ethiopia: A One Health Perspective

**DOI:** 10.4269/ajtmh.24-0401

**Published:** 2024-12-31

**Authors:** Xiaolong Li, Dehao Chen, Song Liang, Jemal Y. Hassen, Sarah L. McKune, Arie H. Havelaar, Jason K. Blackburn

**Affiliations:** ^1^ Department of Environmental and Global Health, College of Public Health and Health Professions, University of Florida, Gainesville, Florida;; ^2^ Emerging Pathogens Institute, University of Florida, Gainesville, Florida;; ^3^ Haramaya University, Dire Dawa, Ethiopia;; ^4^ Center for African Studies, University of Florida, Gainesville, Florida;; ^5^ Global Food Systems Institute, University of Florida, Gainesville, Florida;; ^6^ Department of Animal Sciences, Institute of Food and Agricultural Sciences, University of Florida, Gainesville, Florida;; ^7^ Spatial Epidemiology and Ecology Research Laboratory, Department of Geography, College of Liberal Arts and Sciences, University of Florida, Gainesville, Florida

## Abstract

Increasing attention has focused on health outcomes of *Campylobacter* infections among children younger than 5 years in low-resource settings. Recent evidence suggests that colonization by *Campylobacter* species contributes to environmental enteric dysfunction, malnutrition, and growth faltering in young children. *Campylobacter* species are zoonotic, and factors from humans, animals, and the environment are involved in transmission. Few studies have assessed geospatial effects of environmental factors along with human and animal factors on *Campylobacter* infections. Here, we leveraged *Campylobacter* Genomics and Environmental Enteric Dysfunction project data to model multiple socioenvironmental factors on *Campylobacter* burden among infants in eastern Ethiopia. Stool samples from 106 infants were collected monthly from birth through the first year of life (December 2020–June 2022). Genus-specific TaqMan real-time polymerase chain reaction was performed to detect and quantify *Campylobacter* spp. and calculate cumulative *Campylobacter* burden for each child as the outcome variable. Thirteen regional environmental covariates describing topography, climate, vegetation, soil, and human population density were combined with household demographics, livelihoods/wealth, livestock ownership, and child–animal interactions as explanatory variables. We dichotomized continuous outcome and explanatory variables and built logistic regression models for the first and second halves of the infant’s first year of life. Infants being female, living in households with cattle, reported to have physical contact with animals, or reported to have mouthed soil or animal feces had increased odds of higher cumulative *Campylobacter* burden. Future interventions should focus on infant-specific transmission pathways and create adequate separation of domestic animals from humans to prevent potential fecal exposures.

## INTRODUCTION

*Campylobacter* species are zoonotic bacteria, and the animal reservoirs include poultry, free-living birds, and ruminants, such as cattle and goats, as well as other warm-blooded animals.^1^^,^^2^ Common symptoms of *Campylobacter* infection include fever, diarrhea, and abdominal pain.^3^ In addition to symptomatic infections, asymptomatic *Campylobacter* cases are common, specifically among children and adults in low- and middle-income countries (LMICs).^4^^,^^5^ Evidence from studies in low-resource settings has shown that colonization of *Campylobacter* species acquired at the early stage of child development potentially contributes to environmental enteric dysfunction, malnutrition, and growth faltering in children,^4^^,^^6^^,^^7^ which draws increasing attention to the long-term health effects of *Campylobacter* infections among children younger than 5 years of age in LMICs.

*Campylobacter jejuni* and *Campylobacter coli* have been reported as the two most common species that cause illness in humans.^3^ Traditional methods selectively isolating these two species have resulted in a large body of knowledge specific to their disease burden and clinical manifestations.^8^ However, several *Campylobacter* species other than *C. jejuni/coli* (non-*C. jejuni/coli* species), such as *Campylobacter concisus*, *Campylobacter lari*, *Campylobacter upsaliensis*, and *Campylobacter ureolyticus*, have been recognized as “emerging species” and have shown increasing clinical importance.^9^ Increasing proportions of non-*C. jejuni/coli* species, including a new species “*Candidatus Campylobacter infans*,” were isolated from child stool samples collected in low-resource settings, and several studies have revealed their potential linkage with child stunting.^7^^,^^10^^–^^13^ This evidence shifts our attention toward the genus *Campylobacter* as a whole rather than focusing solely on *C. jejuni/coli* in the context of child health.

The classic “F diagram” (fluids, fields, flies, fingers, fomites, and food) from WHO indicates that adequate sanitation and hygiene can be effective in interrupting the fecal–oral transmission routes of diarrheic pathogens, including *Campylobacter*, in low-resource settings.^14^^,^^15^ Driven by this idea, water, sanitation, and hygiene (WaSH) interventions, including improved pit latrines, handwashing stations, liquid soap, and point-of-use water chlorination, have been developed and implemented in LMICs.^16^^,^^17^ Substantial evidence suggests that adequate WaSH contributes to reducing the risk of childhood diarrheal disease.^18^^,^^19^ However, several recent large-scale randomized, controlled trials have found that WaSH interventions did not significantly reduce the occurrence of diarrhea or other enteric infections.^20^^–^^22^ One possible explanation is that traditional WaSH interventions generally target routes of children’s exposure to human feces but fail to pay adequate attention to children’s exposure to animal feces.^15^^,^^23^ Risk factors for exposing children to animal feces might include livestock ownership, proximity to livestock, cohabitation of humans and animals, etc.

The first year of a child’s life is marked by remarkable developmental milestones, and the differentiation between the first and second 6 months brings about major changes in both biological risk and environmental interaction. In the initial 6 months, breastfeeding plays a crucial role in offering infants essential nutrients and immune protection against pathogens.^24^ However, as the infant transitions into the second 6 months, the introduction of solid foods broadens the child’s nutritional spectrum but also, amplifies exposure to contaminants and pathogens through the food pathway.^25^ Meanwhile, the infant becomes more mobile, gaining the ability to explore the surroundings independently. This newfound mobility empowers infants to explore their environment actively through touching and tasting,^26^ potentially increasing exposure to pathogens through the fecal–oral pathway. This developmental transition underscores the dynamic effects of age on infant’s biological risk and environmental interaction.

Moreover, other environmental factors could impact the nonfood transmission route of *Campylobacter* to humans directly or through animal reservoirs indirectly.^27^^,^^28^ Studies conducted in European countries, the United States, and Canada found campylobacteriosis incidence to be correlated with climatic variables, including temperature and precipitation.^29^^–^^33^ In addition to weather and climate factors, Sanderson et al.^34^ also examined the potential impacts of hydrology and landscape features, like soil type and land use, on the rates of human *Campylobacter* cases in the United Kingdom. This study showed that an increased risk of *Campylobacter* infections was associated with periods of high surface-water flow and catchment areas with cattle/sheep grazing on stagnogley soils. Another similar study conducted in New Zealand linked the risk of infection to a high dairy cattle density.^35^ However, these kinds of studies that investigate the influence of environmental factors have been predominantly conducted in developed countries with a focus on *C. jejuni/coli*. There is a critical knowledge gap in environmental effects on *Campylobacter* infections in LMICs.

Although multiple factors are involved in the transmission pathways of *Campylobacter*, few studies have assessed the combined effects of environmental covariates with other relevant factors from different domains, including humans and animals. Because environmental data often comprise spatial information, such as land cover, precipitation, and topography, geospatial analysis is needed to address the inherent differences in data types between environmental factors and human/animal factors by integrating them within a spatial framework (e.g., spatial regression). This capability to analyze data at different levels from individual households to broader environmental landscapes enhances the depth of risk factor identification. Coupled with the One Health perspective, this kind of analysis will help unravel the complex interplay between human, animal, and environmental factors involved in *Campylobacter* transmissions among children in a low-resource setting.

The longitudinal study of the *Campylobacter* Genomics and Environmental Enteric Dysfunction (CAGED) project conducted in rural eastern Ethiopia aimed to examine the association between *Campylobacter* infection, related reservoirs, and child health outcomes.^36^ In addition to child fecal samples, the CAGED project also collected environmental (e.g., soil and drinking water) and livestock samples for detection and quantification of *Campylobacter*. Results from a previous study showed that the prevalence of *Campylobacter* in all infant stool samples was 64%, and it could increase to as high as 89% as the infants grow older. Moreover, the prevalence in livestock feces ranged from 93% to 99%. In addition to these fecal samples, the household questionnaire used in this project included components on people (e.g., demographics as well as livelihood and wealth); livestock; and the interactions between people, livestock, and the environment. Combined with the child and environmental samples, these data provide an opportunity to investigate the potential combined effects of these risk factors on *Campylobacter* infection in infants.

## MATERIALS AND METHODS

### Study design and protocol.

**Figure 1.**
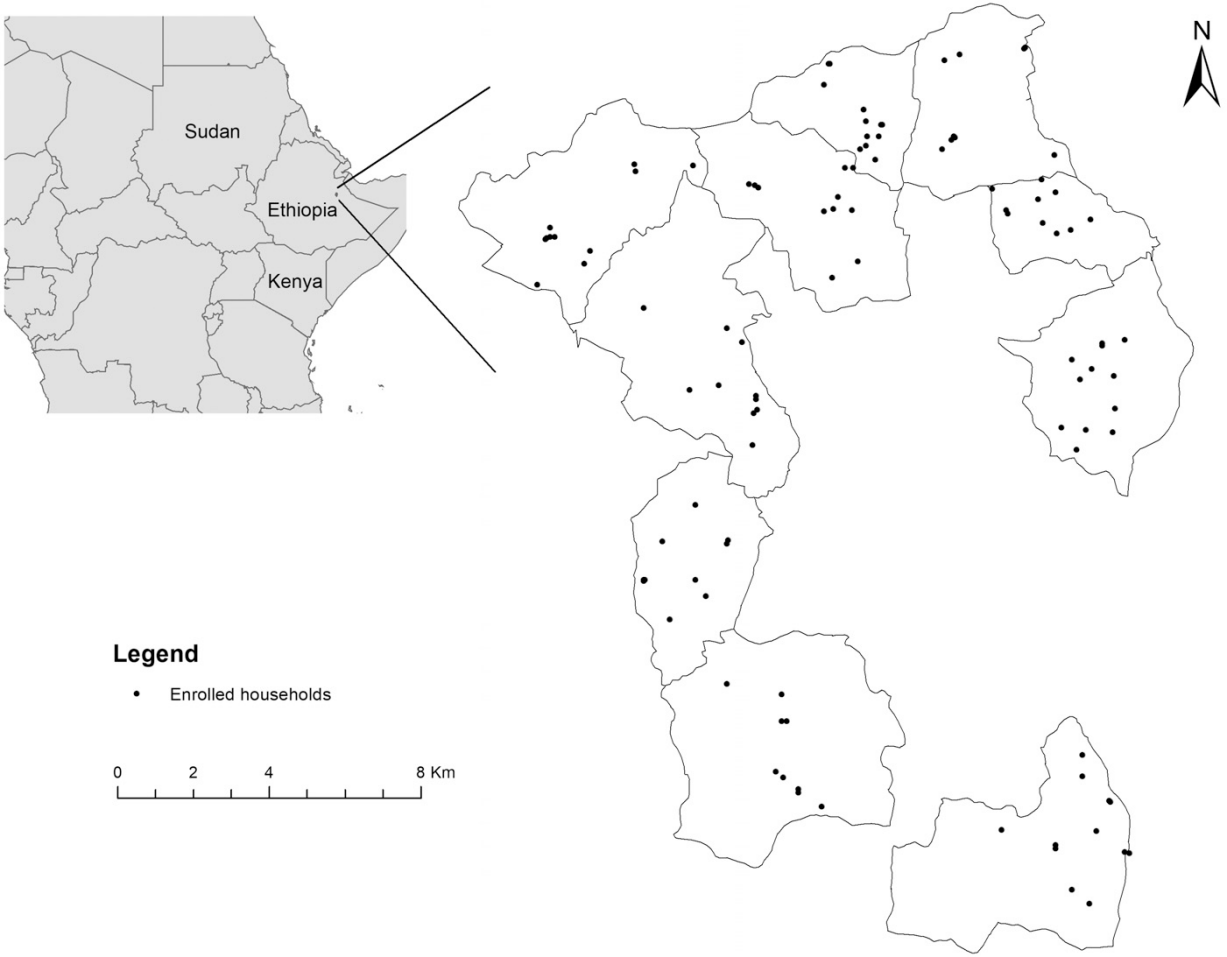
Study area and enrolled households in the *Campylobacter* Genomics and Environmental Enteric Dysfunction project.

A detailed description of the CAGED study design and protocol can be found elsewhere.^36^ Briefly, a total of 106 infants from 10 kebeles (the smallest administrative unit) of Haramaya woreda, Eastern Hararghe Zone, Oromia, Ethiopia (Figure 1) were enrolled at birth and followed until approximately 13 months of age. Written informed consent was obtained from both parents of each participating child in the local language (Afan Oromo). Household information, including demographics, livelihood and wealth, livestock ownership, and child health and nutrition status, was collected through household surveys at baseline and end line along with short surveys conducted monthly. During the study period, child stool samples were collected monthly and tested for *Campylobacter* species. In addition, fecal samples from mothers and siblings of the enrolled children, feces from livestock (i.e., chicken, cattle, goat, and sheep), and environmental samples (soil and drinking water) were collected biannually. DNA extraction, genus-specific TaqMan real-time polymerase chain reaction (PCR; Thermo Fisher Scientific, Waltham, MA), and species-specific Sybr Green real-time PCR were performed afterward to detect, quantify, and characterize *Campylobacter* spp. in these samples.^37^

### Outcome variable.

We calculated the cumulative burden of *Campylobacter* infection for each enrolled child as the outcome variable of interest derived from the Ct values of their fecal samples collected over time. A genus-specific standard curve for expected bacterial load (log genome copies per 50 ng DNA) against the Ct values was first generated using the 16S TaqMan approach.^38^ One milliliter normalized bacterial culture cocktail (including *C. jejuni*, *C. coli*, *Campylobacter hyointestinalis*, *C. lari*, and *Campylobacter fetus*) was used for DNA extraction, and 2 µL extracted DNA was used for quantitative PCR afterward. We used genus-specific primers targeting the *Campylobacter* 16S ribosomal RNA gene (forward primer: GATGACACTTTTCGGAGCGTAA; reverse primer: GCTTGCACCCTCCGTATTA; probe: CGTGCCAGCAGCC-MGB).^38^ The thermocycling conditions consisted of an initial cycle at 95°C for 10 minutes followed by 45 cycles of 95°C for 15 seconds and 55°C for 60 seconds. Nuclease free water and *Salmonella* genomic DNA were used as negative controls. We tested up to 10 *Campylobacter* DNA concentrations and repeated the experiment three times. Then, the Ct values of the tested stool samples were converted to the expected bacterial load using this standard curve. The cumulative *Campylobacter* burden was defined as the average of the expected bacterial loads from available stool samples for each child.^37^

### Explanatory variables: Household surveys.

Human and animal data were derived from household surveys. Demographic data, including child’s sex, mother’s age, and mother’s education level, were selected from the baseline survey along with ownership of livestock (i.e., cattle, goat, sheep, and chicken) and assets (Table 1). To quantify all of the livestock kept by a household, a composite metric (tropical livestock unit) was calculated based on the number of each species of livestock recorded in the baseline household survey.^39^

**Table 1:** Demographics and socioeconomic status of the households enrolled in this study

Variable	*N* = 106*
Sex	
Female	51 (48%)
Male	55 (52%)
Mother’s age (years)	27.0 (22.0–32.0)
Unknown	1
Mother’s education	
No primary education	76 (72%)
Some primary education	29 (28%)
Unknown	1
Livestock ownership	
Cattle	52 (49%)
Goat	60 (57%)
Sheep	48 (45%)
Chicken	53 (50%)
Tropical livestock unit	0.62 (0.20–1.40)

* Values are presented as *n* (percentage) or median (interquartile range).

In the monthly short surveys, we selected variables that reflect interactions between the target child and animals or the environment (e.g., physical contact with animals, crawling in areas with animal droppings, and mouthing of soil or animal feces), diet and nutrition (e.g., consumption of animal source food), feeding practices (e.g., prelacteal feeding, introduction of complementary foods, and consumption of any solid food in the past 24 hours), and use of antibiotics (i.e., being treated with antibiotics in the past month) (Table 2). In addition, two composite variables, minimum dietary diversity (MDD) and household food insecurity access score, were generated according to their respective literatures.^40^^,^^41^

**Table 2:** Variables used for regression modeling and univariate analysis results

Variable	First Period		Second Period	
	OR (CI)*	*P*-Value	OR (CI)	*P*-Value
Child’s sex	0.8 (0.4–1.7)	0.56	2.3 (1.1–5.1)	**0.03**
Mother’s age (years)	1.3 (0.6–2.8)	0.55	0.8 (0.4–1.7)	0.55
Mother’s education	1.2 (0.5–2.9)	0.49	1.1 (0.4–2.6)	0.49
Cattle ownership	0.6 (0.3–1.4)	**0.24** ^†^	2.1 (1.0–4.7)	**0.05**
Goat ownership	1.4 (0.6–3.0)	0.43	0.9 (0.4–1.9)	0.70
Sheep ownership	0.6 (0.3–1.4)	**0.24**	0.5 (0.2–1.2)	**0.12**
Chicken ownership	0.8 (0.4–1.7)	0.56	1.5 (0.7–3.2)	0.33
Tropical livestock unit	1.1 (0.5–2.3)	0.85	1.3 (0.6–2.7)	0.56
Asset	1.3 (0.6–3.0)	0.44	0.8 (0.4–1.8)	0.70
Prelacteal feeding	2.3 (1.0–5.5)	**0.06**	NA	NA
Time of introduction of complementary foods (days)	0.6 (0.3–1.3)	**0.17**	NA	NA
Drinking from a bottle with a nipple in the past 24 hours	0.6 (0.3–1.3)	**0.17**	1.0 (0.5–2.1)	1
Consumption of unpasteurized animal milk in the past 24 hours	1.0 (0.2–4.4)	1	1.4 (0.6–2.9)	0.44
Consumption of any solid food in the past 24 hours	4.5 (1.1–31.1)	**0.04**	1.8 (0.9–4.0)	**0.12**
Consumption of animal source food in the past 24 hours^‡^	NA	NA	0.9 (0.4–1.9)	0.70
Minimum dietary diversity^‡^	NA	NA	0.8 (0.4–1.7)	0.56
Household food insecurity access score	1.3 (0.6–2.7)	0.56	2.3 (1.1–5.1)	**0.03**
Vitamin A supplementation in the past month^§^	NA	NA	1.2 (0.5–2.6)	0.69
Being treated with antibiotics in the past month	1.6 (0.7–3.7)	**0.22**	0.8 (0.4–1.7)	0.55
Contact with animals	3.2 (1.4–7.7)	**0.01**	1.3 (0.6–2.7)	0.56
Crawling in areas with animal feces	3.0 (0.8–14.2)	**0.11**	1.5 (0.7–3.2)	0.33
Mouthing of soil or animal feces	12.1 (2.2–226)	**0.00**	1.5 (0.7–3.2)	0.33
Preventive actions (mother) for mouthing behavior	1.1 (0.5–2.4)	0.77	0.9 (0.4–2.0)	0.84
Elevation (m)	0.6 (0.3–1.3)	**0.17**	0.7 (0.3–1.5)	0.33
Maximum daily land surface temperature, 2021 (°C)	1.3 (0.6–2.7)	0.56	1.1 (0.5–2.3)	0.85
Mean daily land surface temperature, 2021 (°C)	0.9 (0.4–2.0)	0.85	0.8 (0.4–1.7)	0.56
Minimum daily land surface temperature, 2021 (°C)	1.3 (0.6–2.7)	0.56	1.5 (0.7–3.2)	0.33
Maximum 16-day NDVI, 2021	1.5 (0.7–3.2)	0.33	0.9 (0.4–2.0)	0.85
Mean 16-day NDVI, 2021	1.3 (0.6–2.7)	0.56	0.7 (0.3–1.5)	0.33
Minimum 16-day NDVI, 2021	0.8 (0.4–1.7)	0.56	0.5 (0.2–1.1)	**0.08**
Population count 100 × 100 m, 2020	1.2 (0.5–2.5)	0.70	0.3 (0.1–0.6)	**0.00**
Slope (degree)	0.4 (0.2–0.9)	**0.03**	1.3 (0.6–2.7)	0.56
Proportion of clay particles in the fine earth fraction (g/kg)	1.3 (0.6–2.7)	0.56	2.3 (1.1–5.1)	**0.03**
Soil organic carbon content in the fine earth fraction (0.1 g/kg)	0.9 (0.4–1.8)	0.70	1.2 (0.5–2.5)	0.70
Soil pH	0.5 (0.2–1.3)	**0.16**	0.8 (0.3–2.0)	0.64

NA = not applicable; NDVI = normalized difference vegetation index; OR = odds ratio; CI = confidence interval.

* ORs are shown with CIs in parentheses.

^†^ Bold script represents that the *P*-value is less than 0.25 and that the corresponding variable was included as a candidate for the multivariate analysis.

^‡^ Data were not available for the first period as the minimum dietary diversity survey was administered after 6 months of age.

^§^ Only one child had vitamin A supplements for the first period. This variable was excluded from the univariate analysis for the first period.

### Explanatory variables: Environmental covariates.

Thirteen environmental covariates used for ecological niche modeling of the genus *Campylobacter* in a previous analysis^42^ were also included in this study (Table 3). Corresponding values in grids within which the 106 enrolled households were located were extracted from the raster layer of each environmental covariate using the raster package^43^ in R (R Foundation, Vienna, Austria).

**Table 3:** Environmental variables used in this study and their sources

Variable	Median	Data Source
Elevation (m)	2,083.0	WorldPop (https://www.worldpop.org/)
Maximum daily land surface temperature, 2021 (°C)	44.18	MODIS Land Surface Temperature/Emissivity Daily (MOD11A1) v. 6.1
Mean daily land surface temperature, 2021 (°C)	31.17	MODIS Land Surface Temperature/Emissivity Daily (MOD11A1) v. 6.1
Minimum daily land surface temperature, 2021 (°C)	16.58	MODIS Land Surface Temperature/Emissivity Daily (MOD11A1) v. 6.1
Maximum 16-day NDVI, 2021	0.68	MODIS Vegetation Indices (MOD13Q1) v. 6.1
Mean 16-day NDVI, 2021	0.48	MODIS Vegetation Indices (MOD13Q1) v. 6.1
Minimum 16-day NDVI, 2021	0.30	MODIS Vegetation Indices (MOD13Q1) v. 6.1
Population count 100 × 100 m, 2020	6	WorldPop (https://www.worldpop.org/)
Slope (degree)	5.0	WorldPop (https://www.worldpop.org/)
Proportion of clay particles in the fine earth fraction (g/kg)	421.5	SoilGrids v. 2.0 (https://soilgrids.org/)
Soil organic carbon content in the fine earth fraction (0.1 g/kg)	328	SoilGrids v. 2.0 (https://soilgrids.org/)
Soil pH	7.4	SoilGrids v. 2.0 (https://soilgrids.org/)

NDVI = normalized difference vegetation index.

## STATISTICAL ANALYSES

Given that feeding practices (exclusive breastfeeding versus complementary feeding) and the motor ability of infants are most often quite different between the first and second halves of infants’ first year of life, related risk factors, such as probability of exposure to livestock and contaminated environments, are likely to dynamically change over time. Child age is also an important confounding factor for *Campylobacter* infections.^4^^,^^39^ To be consistent with our previous analysis about the prevalence of *Campylobacter* by age groups,^42^ we split the whole study period into two parts, with a cutoff of 177 days of child age, which reflects the boundary between the first two and second two age groups classified in that analysis.

The outcome variable, cumulative *Campylobacter* burden, and all explanatory variables derived from the short household surveys were calculated separately for the two periods. We took the average of the monthly short survey data for each selected short survey variable, which resulted in an average value for the numeric variables or a proportion of being “one” for the binary variables (ordinal variable MDD was dichotomized using a generally accepted cutoff of less than five and greater than or equal to five) during each period. Given the relatively small sample size, a median split approach was applied to dichotomize all continuous variables to improve the model robustness following a previous study.^44^

The purposeful selection of covariates approach was used in this study to choose the candidate variables and determine which to include in the final model.^45^^,^^46^ The likelihood ratio test from logistic regression was performed for each explanatory variable with the outcome variable. The univariate analysis was conducted for the two time periods separately with the same pool of explanatory variables. For the first period, two variables (consumption of animal source food in the past 24 hours and MDD) were excluded from the univariate analysis as these questions were not asked until a child reached 6 months of age. Given that 105 of 106 children did not have vitamin A supplements in the first period, the variable vitamin A supplementation was also excluded from the first-period univariate analysis. Any variables with a *P*-value less than 0.25 were selected as candidates for the following multivariate analysis.

We built a multivariate logistic regression model using an iterative process to select variables. In each iteration, the variable with the highest *P*-value was temporarily removed if it was not significant at the 0.05 alpha level. We checked for changes in the remaining coefficients to assess potential confounding effects. If removing the variable caused a change greater than 20% in any coefficient, indicating confounding, it was added back. This process was repeated until the final model included only significant covariates and potential confounders.

Nagelkerke *R*^2^,^47^ a so-called pseudo-*R*^2^ measure, and the Hosmer and Lemeshow goodness-of-fit test^48^ were used to test the model fit. For a sample size of less than 200 and percentage of success in the outcome variable ranging from 38% to 62%, benchmark values between 0.32 and 0.58 of Nagelkerke *R*^2^ indicate good fit of the model.^49^ The Hosmer and Lemeshow test is a hypothesis test and evaluates if the expected event frequencies from the logistic regression model match the observed event frequencies in subgroups. The area under the receiver operating characteristic curve (AUC) was used to assess the model performance in discriminating the positive results from the negatives.^50^ AUC values range from 0 to 1, and empirically, values between 0.7 and 0.9 are a sign of good predictive performance. Values greater than 0.9 represent an excellent performance.^51^

We first fitted a multivariate model using the data collected from the first period and tested if it was a good fit for the second-period data. If not, a separate model was built for the second period, and effects of the covariates between two periods were evaluated. All of the statistical analyses were performed using R v. 4.1.1.^52^

### Spatial autocorrelation test.

Spatial autocorrelation refers to the correlation within variables across different spatial units.^53^ If the values of a particular variable in nearby locations tend to be similar, a positive spatial autocorrelation exists in this variable, whereas a negative spatial autocorrelation occurs when the values of a variable are more dissimilar than expected with their spatial neighbors. If spatial autocorrelation exists in the residuals of a regression model, it violates the assumption of independent errors and may result in an underestimation of the standard errors of the coefficient estimates of the model.^54^ To test the spatial autocorrelation in the residuals of the logistic models fit in this study, we performed the Moran *I* test on the residuals using the spdep package in R.^55^

## RESULTS

### Demographics and socioeconomic status.

Among the 106 enrolled children, 51.9% were male, and 48.1% were female (Table 1). The mother’s age at baseline ranged from 17 to 43 years, with a median age of 27 years. A high proportion of mothers reported not attending school at any level; only 28% had some primary education. For livestock ownership, 49%, 57%, 45%, and 50% of households owned cattle, goat, sheep, and chicken, respectively. Accordingly, the indicator of tropical livestock units kept by the household ranged from 0 to 5.54, with a median value of 0.62.

### Cumulative *Campylobacter* burden.

**Figure 2.**
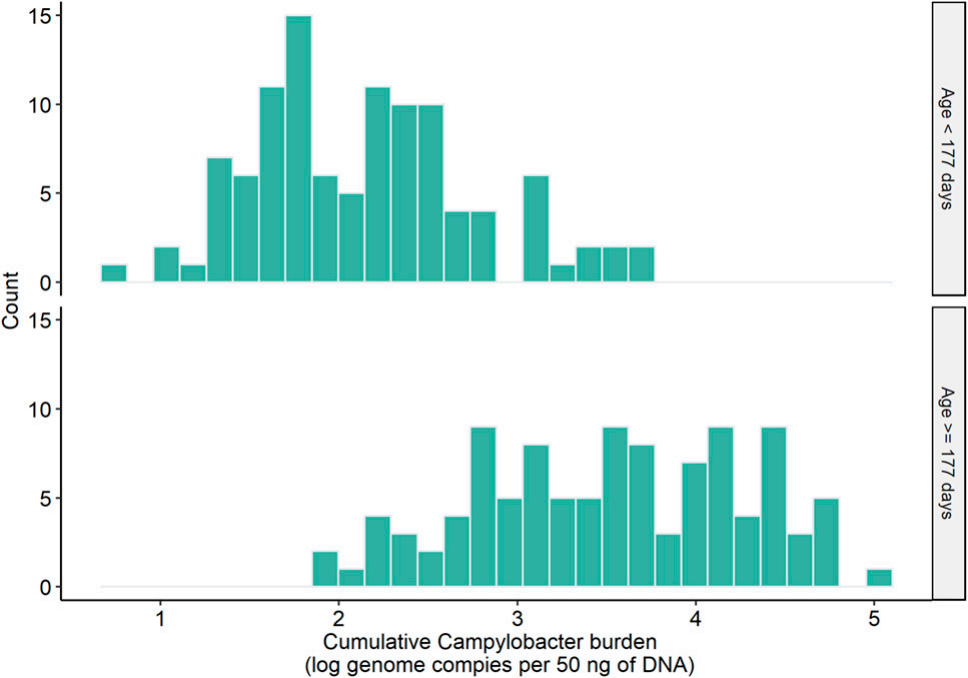
Frequency distribution of the cumulative *Campylobacter* burden for the first and second periods in this study.

The calculated cumulative *Campylobacter* burden for infants in the first period ranged from 0.77 to 3.75 log genome copies per 50 ng DNA, with a median value of 2.10, whereas the minimum and maximum of cumulative *Campylobacter* burden for the second period were 1.95 and 5.06 log genome copies per 50 ng DNA, respectively. The *Campylobacter* burden in the second half year of life (M = 3.52, SD *=* 0.75) was significantly higher than in the first half year of life (M *=* 2.14, SD *=* 0.62) with *t*(210) = 14.7 and *P* <0.01 (Figure 2).

### Univariate analysis.

Based on the *P*-value of the likelihood ratio test with a cutoff of 0.25, 13 and 8 variables were selected as candidate variables for the first period and the second period, respectively (Table 2). Among the candidates, three variables, namely cattle ownership, sheep ownership, and consumption of any solid food in the past 24 hours, were included in the multivariate analysis for both periods. Although child’s sex was not significant at the alpha level of 0.25 in the first period, we still included it in the multivariate analysis given the confounding effect of child’s sex in *Campylobacter* infection reported in previous studies.^4^^,^^7^^,^^39^^,^^56^

### Multivariate analysis.

#### Period 1.

The final logistic regression model for the first period showed that contact with animals, mouthing of soil or animal feces, and drinking from a bottle with a nipple in the past 24 hours were statistically significant at the 0.05 level (Table 4). The direction of the estimated coefficients indicated that children in the first period who had more physical contact with animals and more mouthing of soil or animal feces had greater odds of high cumulative *Campylobacter* burden, whereas drinking from a bottle with a nipple was found to be a protective factor for high *Campylobacter* burden. Sheep ownership was marginally significant in the model and had a negative coefficient estimate. Through the purposeful selection of the covariates process, child’s sex, prelacteal feeding, elevation, and soil pH were identified as potential confounding factors for *Campylobacter* burden.

**Table 4:** Logistic regression model for the cumulative *Campylobacter* burden in the first period

Characteristic	OR	95% CI	*P*-Value
Child’s sex	0.59	0.22–1.51	0.3
Sheep ownership	0.40	0.15–1.01	0.06
Prelacteal feeding	1.77	0.66–4.87	0.3
Contact with animals	3.13	1.11–9.6	**0.04** *
Mouthing of soil or animal feces	12.8	1.80–272	**0.03**
Drinking from a bottle with a nipple in the past 24 hours	0.35	0.12–0.94	**0.04**
Elevation	0.61	0.23–1.60	0.30
Soil pH	0.43	0.14–1.29	0.14
Nagelkerke *R*^2^	0.40	–	–
Hosmer and Lemeshow test	–	–	0.42
AUC	0.79	0.70–0.88	–
Moran *I* statistic	−0.06	–	0.75

AUC = area under the receiver operating characteristic curve; OR = odds ratio; CI = confidence interval.

* Bold represents that the *P*-value is less than 0.05.

**Figure 3.**
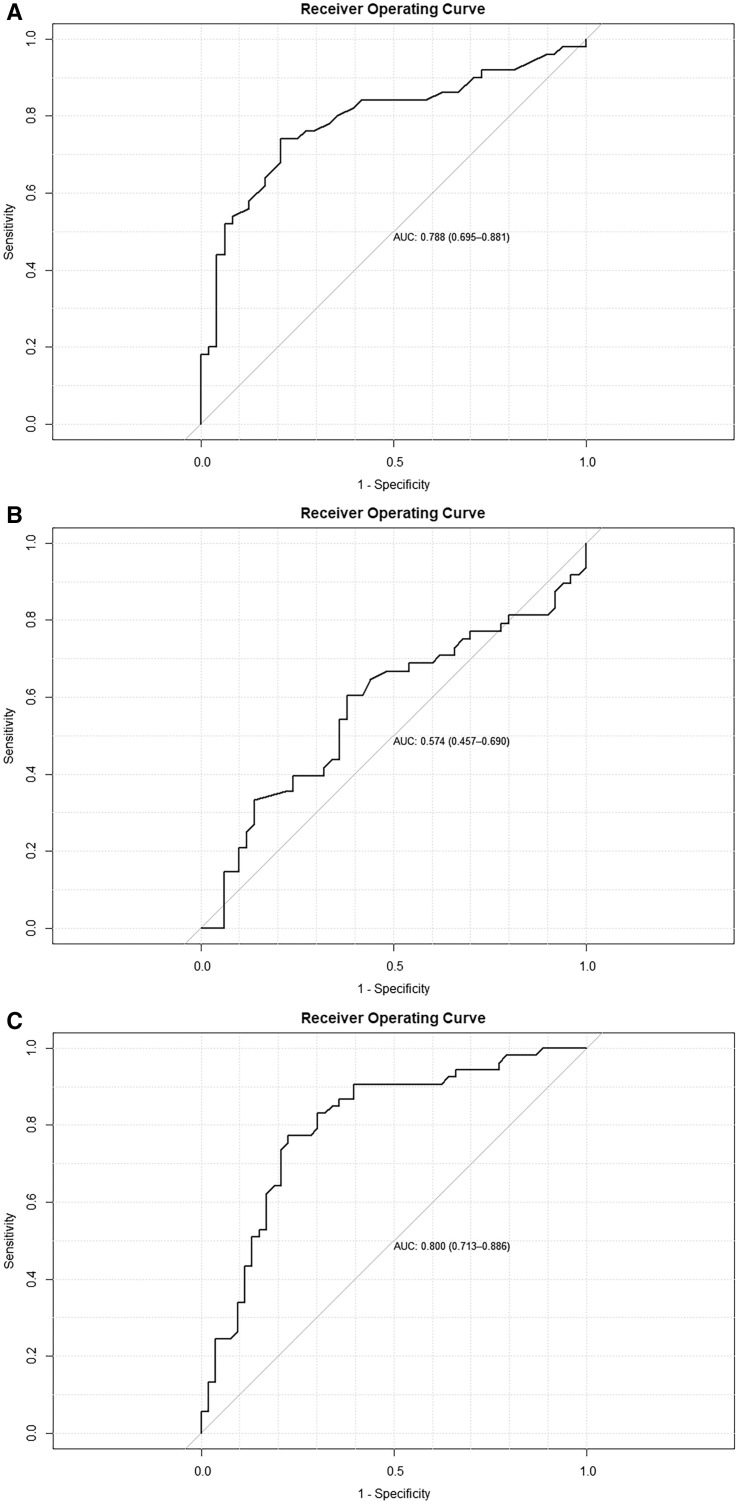
Receiver operating curves for logistic regression models fit in this study. (**A**) Model fit with the first-period data. (**B**) Test of the fit of the first-period model to the second-period data. (**C**) Model fit with the second-period data. AUC = area under the receiver operating characteristic curve.

The Nagelkerke *R*^2^ of the logistic regression model was 0.40, which fell into the benchmark values (0.32–0.58) for good model fit (Table 4). The Hosmer and Lemeshow test also showed that there was no evidence of poor model fit. The model also had a relatively good predictive performance with an AUC of 0.79 (95% CI: 0.70–0.88) (Figure 3A). Moran *I* statistics showed that no spatial autocorrelation existed in the model residuals, suggesting that there was no need to consider a spatial regression model to account for the spatial autocorrelation.

To test if one single model can be obtained for both periods, the second-period data were plugged into the model for the first period as a testing dataset. The Hosmer and Lemeshow test checked the difference between observed data and predicted values, and it showed that this difference was significant (*P* = 0.00), suggesting a poor fit of the model to the second-period data. It was also supported by a low AUC of 0.57 (95% CI: 0.46–0.69) (Figure 3B).

#### Period 2.

Logistic regression models were then fitted using the candidate variables selected from the univariate analysis for the second period, and the final model included eight variables, with child’s sex, cattle ownership, and population density (per 100 m^2^) being statistically significant at the 0.05 level (Table 5). Children being female and living in households that kept cattle had greater odds of high cumulative *Campylobacter* burden, whereas population density showed protective effects on *Campylobacter* burden. Household food insecurity access score, proportion of clay particles in soil, consumption of any solid food in the past 24 hours, and sheep ownership were identified as potential confounding factors for *Campylobacter* burden in the second period.

**Table 5:** Logistic regression model for the cumulative *Campylobacter* burden in the second period

Characteristic	OR	95% CI	*P*-Value
Child’s sex	2.85	1.15–7.47	**0.027** *
Cattle ownership	2.87	1.14–7.70	**0.029**
Sheep ownership	0.49	0.91–1.21	0.12
Household food insecurity access score	1.78	0.69–4.59	0.2
Consumption of any solid food in the past 24 hours	2.01	0.79–5.30	0.15
Minimum 16-day NDVI, 2021	0.43	0.17–1.05	0.069
Population count 100 × 100 m, 2020	0.37	0.15–0.92	**0.033**
Proportion of clay particles in the fine earth fraction	2.12	0.84–5.54	0.12
Nagelkerke *R*^2^	0.34	–	–
Hosmer and Lemeshow test	–	–	0.47
AUC	0.80	0.71–0.89	
Moran *I* statistic	0.014	–	0.37

AUC = area under the receiver operating characteristic curve; NDVI = normalized difference vegetation index; OR = odds ratio; CI = confidence interval.

* Bold represents that the *P*-value is less than 0.05.

The Nagelkerke *R*^2^ for the second model was 0.34, and the Hosmer and Lemeshow test showed that there was no evidence of poor model fit (Table 5). This model had a good predictive performance with an AUC of 0.80 (95% CI: 0.71–0.89) (Figure 3C). Again, there was no spatial autocorrelation existing in the model residuals.

## DISCUSSION

From a One Health perspective, this study identified potential factors involved in the *Campylobacter* transmission pathways between humans, animals, and the environment in a low-resource setting. Child age-specific behaviors and other factors that may increase the risk of children’s exposure to animal feces are largely missing from previous interventions that aimed to mitigate the burden of *Campylobacter* infection or other enteric illnesses among infants and young children in LMICs.^57^ Risk factors of *Campylobacter* infections that have been commonly identified in previous studies conducted among children younger than 5 years of age in low-resource settings are primarily limited to maternal education level, feeding practices, WaSH indicators, and ownership of domestic animals.^4^^,^^7^^,^^58^^,^^59^ However, fewer studies have delved into infant-specific behaviors and the interactions between infants and livestock, which could also contribute to the risk of *Campylobacter* infections.^57^ Considering the dynamic effects of age, particularly distinguishing between infants ages younger than 6 months and those ages 6–12 months, on biological risk and environmental interaction, our study introduces a novel approach. By fitting models and identifying risk factors separately for the first and second 6 months of infancy, we aim to capture the nuanced variations in infection dynamics during these critical developmental stages. This innovative methodology coupled with the exploration of previously overlooked factors using longitudinal data sets our study apart from existing research on *Campylobacter* infection risk factors.

At an early stage of life, infants and young children put nonfood objects in their mouths as one way to explore the surrounding environment.^60^ However, this exploratory behavior could put children at higher risk of contracting zoonotic pathogens if they live in an environment contaminated by livestock and poultry feces. Our results identify mouthing of soil or animal feces as a significant risk factor contributing to a higher cumulative *Campylobacter* burden during the first half of the first year of life. This association can be attributed to the high prevalence of *Campylobacter* spp. in the feces of animals, including domestic livestock and poultry. Results from the laboratory showed that the prevalences of *Campylobacter* in the fecal samples of cattle, sheep, goats, and chicken collected from the CAGED longitudinal study were 99%, 98%, 99%, and 93%, respectively.^37^ In rural areas of LMICs, infants and young children are frequently placed on the ground, sharing space with free-ranging livestock.^57^ As animals defecate on the homestead or sometimes, even inside the homes, enteric pathogens harbored in livestock feces or the soil contaminated by the feces may be ingested by infants through their routine mouthing behaviors while they are on the floor. Ingestion of soil and livestock feces is, therefore, a point of exposure specific to infant and young child behavior. This infant-specific transmission pathway needs to be considered in the future design of intervention strategies for preventing children from having fecal exposure. This finding echoes the collective conclusion from the WaSH Benefits and the Sanitation, Hygiene, Infant Nutrition Efficacy trials: that more effective interventions are needed to reduce the exposure to fecal contaminations in the domestic environments other than the traditional WaSH interventions.^61^

Similarly, physical contact with animals was identified as another risk factor existing in the child–livestock interaction in the first period (before 6 months of age) that increases the exposure of children to animal feces. It is very common for people in rural areas to share living and sleeping quarters with their livestock, which frequently exposes young children in the household to direct contact with livestock. Keeping animals inside human living spaces has been associated with *Campylobacter*-positive child stools in another study in rural Ethiopia,^57^ highlighting the need to design interventions that create adequate separation of domestic animals from the human living spaces to block the transmission pathway through direct contact.

The risks and benefits of raising livestock in smallholder families on child health are intertwined.^23^ On one hand, livestock raised in the household can provide animal source foods, which are seen as the best source of nutrition-rich food for infants and young children. Livestock production is also a source of household income. Increased income from livestock sales can grant families more purchasing power for food, which further helps improve child nutritional status. However, livestock ownership can potentially increase the risk of children’s exposure to *Campylobacter* species. Specifically, free-ranging chickens, which are very common in rural and periurban communities in LMICs, are considered a major source of *Campylobacter* infection.^62^ Their feces can often be found in the home, which potentially increases the risk of *Campylobacter* exposure. According to the responses to our household surveys, chickens were usually kept inside the house (living quarters), on the homestead, or outside the homestead during the day. The frequencies were similar among the three scenarios. However, almost all households owning chickens did not confine their chickens regardless of whether they were kept inside the house or outside. In another analysis of the same population,^44^ we found that keeping chickens unconfined inside the house was associated with a higher *Campylobacter* load over the whole study period. However, that signal was not picked up by the models in the current study after the study period was split into two. Although free-ranging chickens might remain a potential risk factor for *Campylobacter* colonization in rural areas as it is in urban/periurban settings, simple relationships between animal ownership/management and *Campylobacter* colonization were often counterintuitive in our analyses,^44^ suggesting complicated relationships that require more detailed approaches.

In this study, we did not find a significant association between consumption of animal source food and *Campylobacter* burden, but cattle ownership was identified as a significant factor associated with increased odds of having higher cumulative *Campylobacter* burden during the second half of the first year of life. In our study population, when cattle were kept either inside the home or on the homestead but outside the home during the day, 100% (*n* = 27/27) and 93.3% (*n* = 28/30) of households, respectively, confined those cattle. During the night, almost all cattle were kept and tied inside the home. Regarding the purposes of raising cattle, households reported selling livestock for income (44.2%; *n* = 23/52) and consumption of milk (51.9%; *n* = 27/52) as the two major purposes. These data support other findings indicating a strong cultural focus on milk consumption in this area, which may correspond with an underlying risk in the consumption of raw/unpasteurized milk that has been associated with reported outbreaks of campylobacteriosis in high-income countries.^63^^,^^64^ A recent Ethiopian study showed a higher prevalence of *C. jejuni* (16%) in raw milk compared with other dairy products.^65^ Although consumption of raw milk was not identified in our models as a significant risk factor of *Campylobacter* burden in our study population, further work is still needed to unpack this association among children in low-resource settings.

A previous study showed that *Campylobacter* was highly prevalent in livestock feces collected from these households, with prevalences of 99%, 98%, and 93% for cattle, sheep, and chickens, respectively.^37^ It is worth noting that soil samples also had a high *Campylobacter* prevalence of 58%. Even if children living in the household do not have direct contact with livestock, they are still likely to get exposed through other indirect pathways, such as putting soil in their mouth. However, this kind of behavioral data is usually hard to collect through surveys. The Exposure Assessment of *Campylobacter* Infection in Rural Ethiopia project, a sister project of CAGED, was designed to conduct organized child observations for the same cohort as well as collect environmental samples in the households. Results generated from that project will help dissect these potential pathways of children’s exposure to *Campylobacter* species in the future.^66^^,^^67^

Appropriate infant and young child feeding practices could improve child nutritional status, growth, and development.^68^ For infants ages 0–5 months, exclusive breastfeeding is strongly recommended by WHO, whereas bottle-feeding using a bottle with a nipple/teat to feed any liquid or semisolid food is discouraged at this early stage of life because of global concerns that include excessive weight gain, iron depletion, etc.^69^ In addition, a bottle with a nipple is more likely to be contaminated in low-resource settings where inadequate cleaning and disinfection of bottles are more common, which increases the risk of enteric infections.^70^ Lengerh et al.^59^ showed that bottle-feeding was associated with increased odds of *Campylobacter* infection among diarrheic children in northwest Ethiopia. However, in this study, drinking from a bottle with a nipple was shown to be a protective factor for higher cumulative *Campylobacter* burden in the first time period. Our results should not be interpreted as an encouragement of bottle-feeding. One potential explanation could be that this practice is linked to other socioeconomic factors that contribute to reducing the risk of *Campylobacter* infections. A previous study using Ethiopian Demographic and Health Surveys data to examine the determinants of bottle-feeding suggested that women who had a higher education, came from a richer household, and lived in urban areas were more likely to bottle-feed.^69^ The role of the mother’s education level and household wealth status in *Campylobacter* burden was not clear in our analysis with a relatively small sample size, and further work is needed to unpack these potential links.

Influences of environmental and climatic factors have been rarely reported in studies investigating risk factors of *Campylobacter* infections among children in LMICs. Here, we included several environmental covariates previously used to model the prevalence of enteric diseases globally and regionally. Because of the small geography of our study area, most environmental covariates have less heterogeneity to show signals in their association with *Campylobacter* infections. This is one of the limitations in this study. Evaluating these environmental effects on *Campylobacter* infections at a larger scale in LMICs would be a future direction.

We tested the spatial autocorrelation in the model residuals during the model-building process, a step that has seldom been taken in previous work on identifying risk factors of *Campylobacter* infections. We did so not only because we included environmental covariates in the models but also, to consider the potential spatial effects (i.e., spatial dependence and spatial heterogeneity) introduced by georeferenced data.^71^ Neglecting the spatial effects could lead to an inflation of variance in regression estimates and consequently, a less reliable regression model.^72^ Therefore, it would be appropriate for future studies to consider including the spatial autocorrelation test as a core component of the modeling-building process to ensure the reliability and accuracy of the regression model.

Another innovative aspect of this study involves the adoption of bacterial load-based cumulative burden, departing from traditional prevalence measures when dealing with longitudinal data. By calculating the cumulative burden, we gain insight into the persistent impact of *Campylobacter* infections over time, offering a nuanced perspective on disease dynamics. This method enables a comprehensive assessment of *Campylobacter* burden over a certain period and holds promise for advancing longitudinal studies on *Campylobacter* infections. Our previous study indicated that higher *Campylobacter* loads are associated with an increased frequency of diarrhea among children in eastern Ethiopia,^44^ suggesting a potential link between bacterial load and disease occurrence. In addition, results from the Etiology, Risk Factors, and Interactions of Enteric Infections and Malnutrition and the Consequences for Child Health and Development Project (MAL-ED) study suggested that higher *Campylobacter* burden has negatively affected the linear growth (measured by length-for-age *Z* score) of children in low-resource settings.^4^^,^^73^ It is necessary to understand the clinical significance of *Campylobacter* burden, and further investigation is essential to better understand how bacterial burden impacts disease outcomes and to determine whether it could serve as a reliable marker for predicting or managing *Campylobacter*-related illnesses.^73^^–^^75^

## CONCLUSION

In conclusion, our study reveals that factors involved in the interactions between humans (infants), livestock, and home environment impacted the presence of higher cumulative *Campylobacter* burden among infants in eastern Ethiopia. For infants younger than 6 months, being reported to have physical contact with animals and have mouthing of soil or animal feces were identified as risk factors of higher *Campylobacter* burden. Additionally, drinking from a bottle with a nipple was shown to be a protective factor. This result requires additional research to understand and should not be interpreted to encourage bottle-feeding; additional information is required to understand whether there is a direct causal mechanism or whether underlying factors or confounders, such as socioeconomic status or overall household hygiene, might explain the finding. In older infants (ages between 6 and 12 months), being female and living in households with cattle had increased odds of higher *Campylobacter* burden. High population density (potentially linked to urban residency) was identified as a protective factor for this age group. Future interventions should pay more attention to the infant-specific transmission pathway and create adequate separation of domestic animals from humans to prevent infants and young children from potential fecal exposure.

## Data Availability

Deidentified individual participant data will be made available through Dataverse (https://dataverse.org/) after December 31, 2024.

## References

[1] Sahin O Fitzgerald C Stroika S Zhao S Sippy RJ Kwan P Plummer PJ Han J Yaeger MJ Zhang Q, 2012. Molecular evidence for zoonotic transmission of an emergent, highly pathogenic *Campylobacter jejuni* clone in the United States. J Clin Microbiol 50: 680–687. 22189122 10.1128/JCM.06167-11 PMC3295108

[2] Chlebicz A Śliżewska K, 2018. *Campylobacteriosis*, *Salmonellosis*, *Yersiniosis*, and *Listeriosis* as zoonotic foodborne diseases: A review. Int J Environ Res Public Health 15: 863. 29701663 10.3390/ijerph15050863 PMC5981902

[3] Kaakoush NO Castaño-Rodríguez N Mitchell HM Man SM, 2015. Global epidemiology of campylobacter infection. Clin Microbiol Rev 28: 687–720. 26062576 10.1128/CMR.00006-15 PMC4462680

[4] Amour C ; Etiology, Risk Factors, and Interactions of Enteric Infections and Malnutrition and the Consequences for Child Health and Development Project (MAL-ED) Network Investigators, 2016. Epidemiology and impact of *Campylobacter* infection in children in 8 low-resource settings: Results from the MAL-ED study. Clin Infect Dis 63: 1171–1179. 27501842 10.1093/cid/ciw542 PMC5064165

[5] Platts-Mills JA Kosek M, 2014. Update on the burden of *Campylobacter* in developing countries. Curr Opin Infect Dis 27: 444–450. 25023741 10.1097/QCO.0000000000000091 PMC4542018

[6] Lee G Pan W Peñataro Yori P Paredes Olortegui M Tilley D Gregory M Oberhelman R Burga R Chavez CB Kosek M, 2013. Symptomatic and asymptomatic *Campylobacter* infections associated with reduced growth in Peruvian children. PLoS Negl Trop Dis 7: e2036. 23383356 10.1371/journal.pntd.0002036 PMC3561130

[7] Haque MA , 2019. Determinants of *Campylobacter* infection and association with growth and enteric inflammation in children under 2 years of age in low-resource settings. Sci Rep 9: 17124. 31748573 10.1038/s41598-019-53533-3 PMC6868199

[8] François R , 2018. The other *Campylobacters*: Not innocent bystanders in endemic diarrhea and dysentery in children in low-income settings. PLoS Negl Trop Dis 12: e0006200. 29415075 10.1371/journal.pntd.0006200 PMC5819825

[9] Man SM, 2011. The clinical importance of emerging *Campylobacter* species. Nat Rev Gastroenterol Hepatol 8: 669–685. 22025030 10.1038/nrgastro.2011.191

[10] Terefe Y , 2020. Co-occurrence of *Campylobacter* species in children from eastern Ethiopia, and their association with environmental enteric dysfunction, diarrhea, and host microbiome. *Front Public Health* 8: 99. 32351922 10.3389/fpubh.2020.00099 PMC7174729

[11] Bian X , 2020. *Campylobacter* abundance in breastfed infants and identification of a new species in the Global Enterics multicenter study. mSphere 5: e00735-19. 31941810 10.1128/mSphere.00735-19 PMC6968651

[12] Parker CT , 2022. Shotgun metagenomics of fecal samples from children in Peru reveals frequent complex co-infections with multiple *Campylobacter* species. PLoS Negl Trop Dis 16: e0010815. 36194603 10.1371/journal.pntd.0010815 PMC9565744

[13] Garcia Bardales PF , 2022. “Candidatus *Campylobacter* infans” detection is not associated with diarrhea in children under the age of 2 in Peru. PLoS Negl Trop Dis 16: e0010869. 36251729 10.1371/journal.pntd.0010869 PMC9612815

[14] Wagner EG Lanoix JN, 1958. Excreta disposal for rural areas and small communities. Monogr Ser World Health Organ 39: 1–182. 13581743

[15] Penakalapati G Swarthout J Delahoy MJ McAliley L Wodnik B Levy K Freeman MC, 2017. Exposure to animal feces and human health: A systematic review and proposed research priorities. Environ Sci Technol 51: 11537–11552. 28926696 10.1021/acs.est.7b02811 PMC5647569

[16] Sclar GD Penakalapati G Amato HK Garn JV Alexander K Freeman MC Boisson S Medlicott KO Clasen T, 2016. Assessing the impact of sanitation on indicators of fecal exposure along principal transmission pathways: A systematic review. Int J Hyg Environ Health 219: 709–723. 27720133 10.1016/j.ijheh.2016.09.021

[17] Dangour AD Watson L Cumming O Boisson S Che Y Velleman Y Cavill S Allen E Uauy R, 2013. Interventions to improve water quality and supply, sanitation and hygiene practices, and their effects on the nutritional status of children. Cochrane Database Syst Rev 2013: CD009382. 23904195 10.1002/14651858.CD009382.pub2 PMC11608819

[18] Wolf J , 2018. Impact of drinking water, sanitation and handwashing with soap on childhood diarrhoeal disease: Updated meta-analysis and meta-regression. Trop Med Int Health 23: 508–525. 29537671 10.1111/tmi.13051

[19] Freeman MC , 2017. The impact of sanitation on infectious disease and nutritional status: A systematic review and meta-analysis. Int J Hyg Environ Health 220: 928–949. 28602619 10.1016/j.ijheh.2017.05.007

[20] Clasen T , 2014. Effectiveness of a rural sanitation programme on diarrhoea, soil-transmitted helminth infection, and child malnutrition in Odisha, India: A cluster-randomised trial. Lancet GlobHealth 2: e645–e653. 10.1016/S2214-109X(14)70307-9 25442689

[21] Null C , 2018. Effects of water quality, sanitation, handwashing, and nutritional interventions on diarrhoea and child growth in rural Kenya: A cluster-randomised controlled trial. *Lancet Glob Health* 6: e316–e329. 29396219 10.1016/S2214-109X(18)30005-6 PMC5809717

[22] Pickering AJ Djebbari H Lopez C Coulibaly M Alzua ML, 2015. Effect of a community-led sanitation intervention on child diarrhoea and child growth in rural Mali: A cluster-randomised controlled trial. Lancet Glob Heal 3: e701–e711. 10.1016/S2214-109X(15)00144-8 26475017

[23] Chen D Mechlowitz K Li X Schaefer N Havelaar AH McKune SL, 2021. Benefits and risks of smallholder livestock production on child nutrition in low- and middle-income countries. Front Nutr 8: 751686. 34778344 10.3389/fnut.2021.751686 PMC8579112

[24] Hanson LA Wiedermann U Ashraf R Zaman S Adlerberth I Dahlgren U Wold A Jalil F, 1996. Effects of breastfeeding on the baby and on its immune system. Food Nutr Bull 17: 1–5.

[25] Oriá RB Murray-Kolb LE Scharf RJ Pendergast LL Lang DR Kolling GL Guerrant RL, 2016. Early-life enteric infections: Relation between chronic systemic inflammation and poor cognition in children. Nutr Rev 74: 374–386. 27142301 10.1093/nutrit/nuw008 PMC4892302

[26] Gibson E, 1988. Exploratory behavior in the development of perceiving, acting, and the acquiring of knowledge. Annu Rev Psychol 39: 1–42.

[27] Bronowski C James CE Winstanley C, 2014. Role of environmental survival in transmission of *Campylobacter jejuni*. FEMS Microbiol Lett 356: 8–19. 24888326 10.1111/1574-6968.12488

[28] Whiley H van den Akker B Giglio S Bentham R, 2013. The role of environmental reservoirs in human campylobacteriosis. Int J Environ Res Public Health 10: 5886–5907. 24217177 10.3390/ijerph10115886 PMC3863877

[29] Arsenault J Berke O Michel P Ravel A Gosselin P, 2012. Environmental and demographic risk factors for campylobacteriosis: Do various geographical scales tell the same story? BMC Infect Dis 12: 318. 23173982 10.1186/1471-2334-12-318 PMC3570353

[30] Louis VR Gillespie IA O’Brien SJ Russek-Cohen E Pearson AD Colwell RR, 2005. Temperature-driven *Campylobacter* seasonality in England and Wales. Appl Environ Microbiol 71: 85–92. 15640174 10.1128/AEM.71.1.85-92.2005 PMC544220

[31] Kuhn KG Nygård KM Guzman-Herrador B Sunde LS Rimhanen-Finne R Trönnberg L Jepsen MR Ruuhela R Wong WK Ethelberg S, 2020. *Campylobacter* infections expected to increase due to climate change in northern Europe. Sci Rep 10: 13874–13879. 32807810 10.1038/s41598-020-70593-y PMC7431569

[32] Soneja S Jiang C Romeo Upperman C Murtugudde R Mitchell CS Blythe D Sapkota AR Sapkota A, 2016. Extreme precipitation events and increased risk of campylobacteriosis in Maryland, USA. Environ Res 149: 216–221. 27214137 10.1016/j.envres.2016.05.021

[33] Weisent J Seaver W Odoi A Rohrbach B, 2014. The importance of climatic factors and outliers in predicting regional monthly campylobacteriosis risk in Georgia, USA. Int J Biometeorol 58: 1865–1878. 24458769 10.1007/s00484-014-0788-6 PMC4190453

[34] Sanderson RA Maas JA Blain AP Gorton R Ward J O’Brien SJ Hunter PR Rushton SP, 2018. Spatio-temporal models to determine association between *Campylobacter* cases and environment. Int J Epidemiol 47: 202–216. 29069406 10.1093/ije/dyx217 PMC5837245

[35] Spencer SEF Marshall J Pirie R Campbell D Baker MG French NP, 2012. The spatial and temporal determinants of campylobacteriosis notifications in New Zealand, 2001–2007. Epidemiol Infect 140: 1663–1677. 22050713 10.1017/S0950268811002159

[36] Havelaar AH , 2022. Unravelling the reservoirs for colonisation of infants with *Campylobacter* spp. in rural Ethiopia: Protocol for a longitudinal study during a global pandemic and political tensions. BMJ Open 12: e061311. 10.1136/bmjopen-2022-061311 PMC9535169 36198455

[37] Deblais L , 2023. Prevalence and load of the *Campylobacter* genus in infants and associated household contacts in rural eastern Ethiopia: A longitudinal study from the *Campylobacter* Genomics and Environmental Enteric Dysfunction (CAGED) project. Appl Environ Microbiol 89: e0042423. 37310259 10.1128/aem.00424-23 PMC10370295

[38] Platts-Mills JA , 2014. Detection of *Campylobacter* in stool and determination of significance by culture, enzyme immunoassay, and PCR in developing countries. J Clin Microbiol 52: 1074–1080. 24452175 10.1128/JCM.02935-13 PMC3993515

[39] Chen D , 2021. *Campylobacter* colonization, environmental enteric dysfunction, stunting, and associated risk factors among young children in rural Ethiopia: A cross-sectional study from the *Campylobacter* Genomics and Environmental Enteric Dysfunction (CAGED) project. Front Public Heal 8: 615793. 10.3389/fpubh.2020.615793 PMC7862945 33553097

[40] World Health Organization, 2017. Global Nutrition Monitoring Framework: Operational Guidance for Tracking Progress in Meeting Targets for 2025. Available at: https://apps.who.int/iris/bitstream/handle/10665/259904/9789241513609-eng.pdf; jsessionid=5B7CD35139464EA9E9214B4F68A81B5E?sequence=1. Accessed December 1, 2022.

[41] Coates J Swindale A Bilinsky P, 2007. *Household Food Insecurity Access Scale (HFIAS) for Measurement of Food Access: Indicator Guide*. Washington, DC: FHI 360 Food and Nutrition Technical Assistance.

[42] Li X, 2023. Applying Geospatial Approaches to Studying the Epidemiology of Enteric Illnesses at Different Scales. Gainesville, FL: University of Florida.

[43] Hijmans RJ, 2024. Raster: Geographic Analysis and Modeling with Raster Data. Available at: https://rspatial.org/raster. Accessed May 1, 2024.

[44] Chen D , 2024. Campylobacter Colonization and Undernutrition in Infants in Rural Eastern Ethiopia: A Longitudinal Community-Based Birth Cohort Study. Available at: 10.1101/2024.05.21.24307707. Accessed May 30, 2024. PMC11747651 39839388

[45] Bursac Z Gauss CH Williams DK Hosmer DW, 2008. Purposeful selection of variables in logistic regression. Source Code Biol Med 3: 17. 19087314 10.1186/1751-0473-3-17 PMC2633005

[46] Hosmer DW Jr. Lemeshow S Sturdivant RX, 2013. Applied Logistic Regression: Third Edition. Hoboken, NJ: Wiley.

[47] Nagelkerke NJD, 1991. A note on a general definition of the coefficient of determination. *Biometrika* 78: 691–692.

[48] Hosmer DW Lemeshow S, 1980. Goodness of fit tests for the multiple logistic regression model. *Comm Stats Theory Methods* 9: 1043–1069.

[49] Hemmert GAJ Schons LM Wieseke J Schimmelpfennig H, 2018. Log-likelihood-based pseudo-*R*^2^ in logistic regression: Deriving sample-sensitive benchmarks. Sociol Methods Res 47: 507–531.

[50] Hanley JA McNeil BJ, 1982. The meaning and use of the area under a receiver operating characteristic (ROC) curve. Radiology 143: 29–36. 7063747 10.1148/radiology.143.1.7063747

[51] Li H Chen Y Deng S Chen M Fang T Tan H, 2019. Eigenvector spatial filtering-based logistic regression for landslide susceptibility assessment. *ISPRS Int J Geo-Inf* 8: 332.

[52] R Core Team, 2021. R: A Language and Environment for Statistical Computing. Vienna, Austria: R Foundation for Statistical Computing.

[53] Getis A, 2007. Reflections on spatial autocorrelation. Reg Sci Urban Econ 37: 491–496.

[54] Lichstein JW Simons TR Shriner SA Franzreb KE, 2002. Spatial autocorrelation and autoregressive models in ecology. Ecol Monogr 72: 445–463.

[55] Bivand R, 2022. R packages for analyzing spatial data: A comparative case study with areal data. Geogr Anal 54: 488–518.

[56] Strachan NJC Watson RO Novik V Hofreuter D Ogden ID Galán JE, 2008. Sexual dimorphism in campylobacteriosis. Epidemiol Infect 136: 1492–1495. 18062834 10.1017/S0950268807009934 PMC2870750

[57] Budge S Barnett M Hutchings P Parker A Tyrrel S Hassard F Garbutt C Moges M Woldemedhin F Jemal M, 2020. Risk factors and transmission pathways associated with infant *Campylobacter* spp. prevalence and malnutrition: A formative study in rural Ethiopia. PLoS One 15: e0232541. 32384130 10.1371/journal.pone.0232541 PMC7209302

[58] Diriba K Awulachew E Anja A, 2021. Prevalence and associated factor of *Campylobacter* species among less than 5-year-old children in Ethiopia: A systematic review and meta-analysis. Eur J Med Res 26: 2. 33390175 10.1186/s40001-020-00474-7 PMC7780653

[59] Lengerh A Moges F Unakal C Anagaw B, 2013. Prevalence, associated risk factors and antimicrobial susceptibility pattern of *Campylobacter* species among under five diarrheic children at Gondar University Hospital, northwest Ethiopia. BMC Pediatr 13: 82. 23694714 10.1186/1471-2431-13-82 PMC3663702

[60] Ruff HA Saltarelli LM Capozzoli M Dubiner K, 1992. The differentiation of activity in infants’ exploration of objects. Dev Psychol 28: 851–861.

[61] Pickering AJ , 2019. The WASH benefits and SHINE trials: Interpretation of WASH intervention effects on linear growth and diarrhoea. *Lancet Glob Health* 7: e1139–e1146. 31303300 10.1016/S2214-109X(19)30268-2

[62] Schiaffino F Trigoso DR Colston JM Olortegui MP Shapiama Lopez WV Garcia Bardales PF Pisanic N Davis MF Yori PP Kosek MN, 2021. Associations among household animal ownership, infrastructure, and hygiene characteristics with source attribution of household fecal contamination in peri-urban communities of Iquitos, Peru. Am J Trop Med Hyg 104: 372–381. 33146117 10.4269/ajtmh.20-0810 PMC7790101

[63] Wulsten IF Galeev A Stingl K, 2020. Underestimated survival of *Campylobacter* in raw milk highlighted by viability real-time PCR and growth recovery. Front Microbiol 11: 1107. 32625171 10.3389/fmicb.2020.01107 PMC7311638

[64] Davys G Marshall JC Fayaz A Weir RP Benschop J, 2020. Campylobacteriosis associated with the consumption of unpasteurised milk: Findings from a sentinel surveillance site. Epidemiol Infect 148: e16. 32014081 10.1017/S0950268819002292 PMC7019552

[65] Admasie A Eshetu A Tessema TS Vipham J Kovac J Zewdu A, 2023. Prevalence of *Campylobacter* species and associated risk factors for contamination of dairy products collected in a dry season from major milk sheds in Ethiopia. Food Microbiol 109: 104145. 36309427 10.1016/j.fm.2022.104145

[66] Deblais L , 2024. Assessing Fecal Contamination from Human and Environmental Sources Using Escherichia coli as an Indicator in Rural Ethiopian Households—A Study from the EXCAM Project. Available at: 10.1101/2024.08.21.24312392. Accessed August 30, 2024. PMC11743629 39835307

[67] Wang Y , 2024. Quantitative Multi-Pathway Assessment of Exposure to Fecal Contamination for Infants in Rural Ethiopia. Available at: 10.1101/2024.08.29.24312786. Accessed September 1, 2024.

[68] World Health Organization, 2008. Indicators for Assessing Infant and Young Child Feeding Practices: Conclusions of a Consensus Meeting Held 6–8 November 2007 in Washington, DC. Geneva, Switzerland: WHO.

[69] Belay DG Getnet M Akalu Y Diress M Gela YY Getahun AB Bitew DA Terefe B Belsti Y, 2022. Spatial distribution and determinants of bottle feeding among children 0–23 months in Ethiopia: Spatial and multi-level analysis based on 2016 EDHS. BMC Pediatr 22: 120. 35264134 10.1186/s12887-022-03181-w PMC8905773

[70] Rothstein JD Mendoza AL Cabrera LZ Pachas J Calderón M Pajuelo MJ Caulfield LE Winch PJ Gilman RH, 2019. Household contamination of baby bottles and opportunities to improve bottle hygiene in peri-urban Lima, Peru. Am J Trop Med Hyg 100: 988–997. 30834885 10.4269/ajtmh.18-0301 PMC6447096

[71] Anselin L Getis A, 1992. Spatial statistical analysis and geographic information systems. Ann Reg Sci 26: 19–33.

[72] Bertazzon S Olson S Knudtson M, 2010. A spatial analysis of the demographic and socio-economic variables associated with cardiovascular disease in Calgary (Canada). *Appl Spatial Anal* 3: 1–23.

[73] Rogawski ET , 2018. Use of quantitative molecular diagnostic methods to investigate the effect of enteropathogen infections on linear growth in children in low-resource settings: Longitudinal analysis of results from the MAL-ED cohort study. Lancet Glob Health 6: e1319–e1328. 30287125 10.1016/S2214-109X(18)30351-6 PMC6227248

[74] Liu J , 2016. Use of quantitative molecular diagnostic methods to identify causes of diarrhoea in children: A reanalysis of the GEMS case-control study. Lancet 388: 1291–1301. 27673470 10.1016/S0140-6736(16)31529-X PMC5471845

[75] Platts-Mills JA McQuade ETR, 2023. Assigning pathogen etiology for childhood diarrhea in high-burden settings: A call for innovative approaches. J Infect Dis 228: 814–817. 37504374 10.1093/infdis/jiad277

